# Addressing the rising rates of gonorrhea and drug-resistant gonorrhea: There is no time like the present

**DOI:** 10.14745/ccdr.v45i23a02

**Published:** 2019-02-07

**Authors:** M Bodie, M Gale-Rowe, S Alexandre, U Auguste, K Tomas, I Martin

**Affiliations:** 1 Centre for Communicable Diseases and Infection Control, Public Health Agency of Canada, Ottawa, ON; 2 Bacterial Pathogens Division, National Microbiology Laboratory, Public Health Agency of Canada, Winnipeg, MB

**Keywords:** *Neisseria gonorrhoeae*, antimicrobial resistance, clinician, public health care provider, *N. gonorrhoeae*, gonococcal

## Abstract

Increasing rates of gonococcal (GC) infection and antimicrobial resistant (AMR) GC, are a serious public health concern for Canada and around the world. Previously recommended treatments are ineffective against many of the gonorrhea strains circulating today. The current recommendation for combination therapy is now being threatened by globally emerging and increasingly resistant strains. It is important that coordinated efforts be made now to ensure these new global strains do not become established in Canada. Otherwise, we will be faced with the possibility of persistent GC infection which can lead to pelvic inflammatory disease, infertility and chronic pelvic pain in women; and epididymitis in men. The presence of GC can also increase the risk of HIV acquisition and transmission.

There are a number of reasons why we are facing this public health threat. GC infection is often asymptomatic and it is highly transmissible. People may hesitate to seek testing (or to offer testing). Treatment is complex: recommendations vary by site of infection and risk of resistance. Sexual contact during travel is an important source of imported emerging resistant global strains. The new screening and diagnostic Nucleic Acid Amplification Test (NAAT) is excellent but has decreased the number of cultures being done and therefore our capacity to track AMR-GC.

There are four key actions that clinicians and front-line public health professionals can take to stem the increase in rates of GC and drug resistant GC. First, normalize and increase GC screening based on risk factors and emphasize the need for safer sex practices. NAAT is useful for screening, but culture is still needed for extra-genital sites. Second, conduct pretravel counselling and include a travel history as part of the risk assessment. Third, use culture along with NAAT to establish the diagnosis and follow up for test-of-cure. Finally, refer to the most current *Canadian Guidelines on Sexually Transmitted Infections* or provincial/territorial recommendations on combination therapies for patients and their contacts as recommendations may have changed in response to evolving AMR-GC trends.

## Introduction

Increasing antimicrobial resistance (AMR) in *Neisseria gonorrhoeae*, seen both domestically and internationally, combined with rising rates of gonococcal (GC) infection, are a serious public health concern. Although GC is treatable, global rates continue to rise. The World Health Organization (WHO) estimates that 78 million people are infected with GC annually, as a result of decreased condom use, increased urbanization and travel, poor infection detection rates and inadequate or failed treatment (1).

The Public Health Agency of Canada (PHAC) provides annual national data on GC infection rates (2), laboratory surveillance of AMR-GC (3), and recommendations on the prevention, early detection and treatment of gonorrhea in the *Canadian Guidelines on Sexually Transmitted Infections* (CGSTI) (4). Due to the documented increase in rates of gonorrhea and AMR-GC, PHAC is identifying the need for concerted clinical and public health action.

Rates of gonorrhea have been increasing steadily over the last few years. The number of cases reported in 2016 was more than double the number reported in 2010 (rising from 33.5 to 65.4 per 100,000 population), corresponding to a 95% increase in rates. Males accounted for at least 56% of all cases diagnosed. The most commonly affected age group was 15 to 39 year olds; they comprised 82% of reported cases of gonorrhea, although they represented only 33% of the total population. However, the rates among those aged 40+ have doubled in the past ten years (2).

*N. gonorrhoeae* now shows resistance to six previously recommended treatment options: sulfonamides, penicillins, earlier generation cephalosporins, tetracyclines, macrolides, and fluoroquinolones (5). The current options for first-line therapy are the third generation extended-spectrum cephalosporins (cefixime, ceftriaxone) and azithromycin. For the purposes of tracking resistance, AMR-GC has been classified as either multidrug-resistant gonococci (MDR-GC) or extensively drug-resistant gonococci (XDR-GC). MDR-GC is defined as a GC strain with decreased susceptibility/resistance to one currently recommended therapy (cephalosporin or azithromycin) plus resistance to at least two other antimicrobials. XDR-GC is defined as a GC strain with decreased susceptibility/resistance to two currently recommended therapies (cephalosporin and azithromycin) plus resistance to at least two other antimicrobials (6,7).

In Canada and globally, isolates are exhibiting decreased susceptibilities to the extended-spectrum cephalosporins and increasing resistance to azithromycin, and treatment failures have been reported (8–11). In this issue, Martin et al. identify that between 2012 and 2016, the proportion of MDR-GC isolates increased from 6.2% to 8.9% and that XDR-GC was rare (0.1% over the same 4-year period) (7). The resistance profile varied by antibiotic. The proportion of isolates with decreased susceptibility to cephalosporins declined between 2012 and 2016 (from 5.9% to 2.0%). During the same period, however, azithromycin resistance increased from 0.8% to 7.2% with some regional variability: the rates were highest in Quebec (18%) and Ontario (5%) (7).

While the low rates of XDR-GC are encouraging, there are new concerns about MDR-GC in Canada. Since the Martin study, two travel related cases were reported with ceftriaxone-resistant strains of GC not previously seen in Canada. The first case was an asymptomatic female whose partner had travelled to China and Thailand (11,12). The second case was a male who had a sexual contact with someone who was visiting from Southeast Asia. Isolates from both cases were genetically liked to *N.gonorrhoeae* strain FC428, first identified in Japan (2015). Both cases were eventually successfully treated; the first with high dose cefixime and azithromycin (11,12) and the second with gentamicin and azithromycin (*Personal communication, Petra Smyczek, Alberta Health Services, July 31, 2018).* This strain has now been identified and characterized in a number of other countries through travel (e.g., Australia, France, Ireland) (11–14). Globally, cases of XDR-GC with High-Level azithromycin (HL-Az) resistance have been reported in the United Kingdom and Australia (15–17); some of these cases were also associated with travel to Southeast Asia.

It is important that efforts be made now to curtail the progression of AMR-GC in general, and to ensure that novel resistant strains (both MDR-GC and XDR-GC) not become established in Canada. Failure to prevent this could add to the already significant morbidity caused by GC infection.

Undiagnosed/untreated GC infection is not benign. In women, it can lead to pelvic inflammatory disease (PID), ectopic pregnancy, chronic pelvic pain and infertility. In men, it can cause epididymitis (pain and swelling of the testicles). The presence of GC can also increase the risk of HIV acquisition and transmission (18–20).

How has this treatable infection become such a public health threat? The objective of this article is to identify how this happened and more importantly, what we can do about it. We make the case that there are four actions that every clinician and front-line public health professional can do to stop the rising GC rates and prevent emerging MDR-GC and XDR-GC from becoming established in Canada.

## How did this happen?

There are at least seven factors that have led to this situation.

### Gonorrheal infections are often asymptomatic

Women are usually asymptomatic or have only minor symptoms that can easily be ascribed to something else (21–23). Men with urethral gonococcal infection usually have symptoms, but in both genders, rectal and pharyngeal infections are more likely to be asymptomatic (24–26).

### Gonorrhea is highly transmissible

Gonococcal infection spreads easily. The estimated transmission rate from a single sexual encounter is 50% to 60% from an infected man to an uninfected woman and 20% from an infected woman to an uninfected man (27). This combination of a highly infectious organism along with a lack of symptoms results in a high rate of onward transmission.

### Sexual contact during travel is not uncommon

It is well documented that travel is associated with sexual risk-taking (28–30). Travel is often described as a temporary escape from social expectations in everyday life, contributing to a sense of anonymity and engaging in behaviours that may not be acceptable at home (30–32). Estimated pooled prevalence of travel-related, casual sex among international travellers is approximately 20% to 34% (30,33). Gay, bisexual, or men who have sex with men (gbMSM) are 2–3 times more likely to report a new sexual partner while overseas (34–36) and the proportion of gbMSM having unprotected anal intercourse with a casual partner abroad ranges from 22% to 60% (30).

The recent reports of the novel strain FC428 in travel-related AMR-GC cases in Canada reminds us that certain parts of the world, most particularly Southeast Asia, have seen the emergence of novel resistant strains (MDR and XDR) that pose a risk to Canadians.

### People are reticent to seek or offer testing

Unfortunately, there are multiple reasons why people may not seek testing. Individuals tend to underestimate their personal risk. They may perceive that sexually transmitted infections (STIs) are not serious, may be fearful of invasive procedures, or self-conscious about a genital examination. There may be other barriers, such as perceived or anticipated attitudes of health care providers and clinic staff, which can result in individuals feeling judged and discriminated against (37,38). For example, it has been reported that only 49% to 70% of gbMSM have disclosed their sexual orientation to physicians (39,40). Lastly, social barriers exist when individuals fear social condemnation (stigma) with STI testing (37).

Health care providers may not offer testing. Those who don’t deal frequently with screening/management of STI may lack knowledge of when and how to test for STI, as well as how to treat a positive result. Discomfort with taking a sexual history and performing genital exams, and a lack of time due to competing medical priorities, have been cited as barriers to STI testing (41–44).

### Treatment recommendations can change and are complex

Treatment recommendations keep changing to keep up with the changing resistance profiles. For example, based on rising resistance levels, PHAC changed its first-line treatment recommendations to combination therapy in 2013 (i.e., ceftriaxone/cefixime plus azithromycin). In 2017, PHAC issued an additional alternative treatment recommendation (gentamicin and azithromycin) for GC infections (45). Not all clinicians may be informed of these changing recommendations or they may follow other recommendations.

Treatment recommendations are also complex. Drugs and dosages may differ by the site of the infection or the sexual activity of the person affected. This means that treatment prescribed for an uncomplicated genital infection will not be adequate to treat a pharyngeal infection, which is more difficult to eradicate.

### Canada has lost some capacity to track resistant gonorrhea

The use of Nucleic Acid Amplification Test (NAAT) has largely been seen as an advance in GC diagnostics, mainly because of its ease of use (it can be done on urine) and its high sensitivity—up to 100% in some cases. One of the unintended consequences of NAAT is that fewer cultures are being taken as a result, and cultures are currently required for antimicrobial susceptibility testing. In 2016, for example, of the 23,708 cases reported, only about 19% were cultured (46). This means that direct AMR data was only available on approximately one-fifth of GC cases in Canada.

### Resistant strains have a competitive advantage over non-resistant strains

MDR and XDR strains of any bacterial infection can spread quickly. Since they are difficult to treat, transmission can continue unabated.

## Recommendations for action

In light of the recent development of novel strains resistant to our remaining first-line treatment options, there are four actions that clinicians and front-line public health professionals can do.

### 1. Normalize and increase screening and promote safer sex practices

There are several ways that health care providers can reduce the hesitations around testing. One key strategy is to normalize it and offer screening for GC—and other sexually transmitted and blood-borne infections (STBBI) with similar routes of transmission—in the course of routine medical care. Using urine NAAT for screening can reduce barriers as it is less invasive for the patient and less time consuming for the clinician.

Be alert for opportunities to have a conversation about STI risks, safer sex practices and the benefits of screening. Perform a risk assessment and offer STI screening to individuals seeking contraceptive advice or to individuals who have a new partner. Although young adults are at the highest risk for STIs, middle aged and older adults may also be at risk and could benefit from screening. Emphasize the need for consistent and correct use of condoms.

The Canadian Public Health Association has developed an excellent resource on best practices when discussing sensitive issues regarding sexual health, substance use and STBBIs to assist providers in the course of assessing risk and/or educating patients (47). This resource may reduce barriers related to discomfort with discussing risk behaviours. A brief risk assessment can be used to quickly identify or rule out major risk factors associated with the increased risk of STIs. Any patient whose current or past history identifies a potential risk factor for STI should be asked to complete a more detailed history.

#### Screening recommendations

 

Screening should be offered based on risk. Major risk factors for GC infection include:

- A history of STI (including HIV infection)
- A partner who has been diagnosed with GC
- Sexually active youth less than 25 years of age (due to the high burden of disease in this age group)
- Unprotected sex
- Multiple partners
- gbMSM
- Having a new sex partner in the context of travel

Screening is particularly important during pregnancy, as untreated infection can cause serious illness in the newborn. The Canadian Paediatric Society issued a recommendation against prophylaxis for ophthalmia neonatorum (48). All pregnant women at risk should be screened at the first prenatal visit or at the time of delivery if not previously screened. In serodiscordant couples, the presence of GC, chlamydia or other STI in either partner can increase the risk of HIV transmission (18–20).

#### NAAT and culture

 

For screening asymptomatic males, first-void urine for NAAT is the test of choice (49). For screening asymptomatic females, vaginal swabs are preferred and may be self-collected. Cervical swabs for NAATs can be taken. Urine-based NAAT is ideal when a pelvic examination is not indicated or is refused.

Depending on the history and the clinical situation, it may be appropriate to take samples from multiple (i.e., all exposed) anatomical sites. Culture remains the preferred test for screening of extra-genital (pharyngeal and rectal) infections (a validated NAAT may be used if culture is not available). As pharyngeal infections are usually asymptomatic, using culture to screen those with a history of performing oral sex is extremely important.

### 2. Conduct pretravel counselling and take a travel history

Health care providers need to counsel travellers prior to travel on the need for safer sex practices. Depending on the destination, it may be appropriate to discuss specifically the risk of AMR-GC infection.

When an individual presents with symptoms or a possible exposure to an STI, the assessment should include a travel history. If the travel risk assessment identifies unprotected sexual exposure during travel there should be a heightened index of suspicion for potential AMR-GC infection, and more specifically, a resistant strain not currently circulating in Canada.

### 3. Increase diagnosis and follow-up with a test-of-cure

Cultures are important for the diagnosis of symptomatic patients, and critical for gaining information on antimicrobial resistance testing. However, NAAT is important too, as it is the most sensitive diagnostic test. When a patient has signs and/or symptoms consistent with GC, the use of culture together with NAAT is extremely useful. This permits antimicrobial susceptibility testing and identification of AMR strains, while the use of highly sensitive NAAT reduces the number of missed diagnoses.

Samples should be taken from all exposed sites. If symptomatic patients are given empiric therapy (i.e., before test results are available), specimens should be obtained prior to treatment. Due to high rates of concomitant infection, specimens should be taken for the diagnosis of both GC and chlamydial infections (50).

#### Test for urogenital gonorrhea

 

In symptomatic men with urogenital infections, a urethral swab for gram stain and culture should be obtained when possible. When culture testing is not available, urine NAAT can be used (51). In symptomatic women, collect a cervical or vaginal swab for culture and for NAAT. The use of culture is important and is strongly recommended, especially under certain circumstances (e.g., to evaluate pelvic inflammatory disease and in pregnancy). Vaginal swabs or urine are suitable samples for NAAT. In patients with urethral symptoms, a urethral swab for culture can also be used.

#### Test for extra-genital gonorrhea

 

For sexually active individuals with extra-genital signs and symptoms or with a history of performing oral sex or having receptive anal intercourse, collect pharyngeal and rectal specimens for testing. Culture remains the preferred testing method for diagnosis of extra-genital infections, which are often asymptomatic. If culture is not available, check whether your laboratory has done an in-house laboratory validation on clinical specimens (i.e., pharyngeal and rectal) other than the manufacturer’s recommended specimen type (i.e., urine). If it has, this “validated” NAAT may be used for extra-genital specimens.

#### Follow-up with a test-of-cure

 

Take follow-up cultures for test-of-cure from all positive sites 3–7 days after the completion of treatment. If NAAT is the only option for test-of-cure, take this 2–3 weeks after completion of treatment to avoid false-positive results.

Tests-of-cure are particularly important for:

- Pharyngeal infections
- Pregnant women
- High risk of AMR-GC (i.e., diagnosed in partner or following travel and sexual contact in an area with a high burden of AMR-GC)
- Alternative treatment regimens when ceftriaxone was the first-line treatment but intravenous therapy was not possible
- Persistent signs/symptoms

Repeat screening six months post-treatment is recommended due to the risk of reinfection.

### 4. Provide up-to-date combination therapy to patients and their contacts

Treat all patients with GC with combination therapy (52). The use of two antimicrobials with different mechanisms of action is thought to improve treatment efficacy as well as to prevent or potentially delay the emergence and spread of AMR-GC. In order to prevent development of AMR, monotherapy with azithromycin, 2 grams should be avoided unless there is no other option. Prompt and appropriate treatment of infected individuals, and all sexual partners from the preceding 60 days, is essential to prevent the spread of infection. The local public health professionals can assist with contact tracing and notification as needed.

All individuals should be treated according to current recommendations (45). These recommendations are also available and accessible through the CGSTI mobile application. This application provides quick and convenient access to up-to-date Canadian guidelines on the diagnosis and management of STI. It is available for free for Apple™ and Android™ devices and can be accessed via the CGSTI website (4).

#### Treatment recommendations

 

Recommended combination therapy varies depending on site of infection and probability of resistance. In particular, the CGSTI differentiates between treatment options for uncomplicated anogenital gonococcal infections, and the treatment of pharyngeal infections (in all adults) as well as infections among gbMSM.

Check the CGSTI or STI guidance from local or provincial/territorial (P/T) public health for details on treatment recommendations (4). Local or P/T guidance should be followed when treatment choices have been informed by local or P/T resistance data.

When cephalosporins are contraindicated because of allergy or resistance, the CGSTI recommend treatment with alternative combination therapy regimens that include gentamicin (4).

If persistent infection is suspected following treatment, both culture and antimicrobial susceptibility testing should be performed to verify treatment failure and assess effective and appropriate treatment options. Consultation with an infectious disease specialist is warranted.

These four key recommendations for action are summarized in **Table 1**.

**Table 1:** Four key recommendations needed to preserve options for remaining first-line treatment of antimicrobial resistant gonorrhea

Recommendations	Details
Normalize and increase screening and promote safer sex practices	To reduce barriers and associated stigma, look for opportunities during routine medical care to have a conversation about STI risks, safer sex practices and the benefits of screening Samples should be taken from all sites of exposure, to increase diagnosis and ensure appropriate treatment is provided
Conduct pretravel counselling Include a travel history in your risk assessment	Counsel travellers on the importance of safer sex practices while travelling; depending on the destination, it may be appropriate to discuss the risk of AMR-GC infection specifically If there is a history of unprotected sexual exposure during travel, maintain a heightened index of suspicion for potential AMR-GC infection, and more specifically, a globally emerging resistant strain not currently circulating in Canada
Increase the use of cultures for diagnosis and test-of-cure	NAAT is convenient and highly sensitive and can increase the diagnosis of GC. Culture provides information on antimicrobial susceptibilities prior to treatment and is critical for improved public health monitoring of antimicrobial resistance patterns and trends When signs and/or symptoms are consistent with gonococcal infection, the use of culture along with NAAT is extremely important
Provide up-to-date combination therapy for patients and their contacts	Due to increasing antimicrobial resistance, combination therapy is the standard of care choice of combination therapy should be guided by infection site and patient history. AMR resistance patterns may show regional variation Consult the CGSTI or your jurisdiction’s STI guidance for details on treatment recommendations Treatment of all sexual contacts from the previous 60 days is essential. Local public health professionals can assist with contact tracing and notification as needed

Abbreviations: AMR, antimicrobial resistance; CGSTI, Canadian Guidelines on Sexually Transmitted Infections (4); GC, gonococcal; NAAT, nucleic acid amplification testing; STI, sexually transmitted infection

## Discussion

Rates of GC and AMR-CG infections are increasing, both domestically and internationally, and represent a serious public health concern. Clinicians and front-line public health professionals are well placed to proactively screen and treat patients testing positive for GC or AMR-GC, and counsel all those at risk, on the risks of STIs and travel. Cultures are needed for diagnosis when possible, and to assess treatment effectiveness, to prevent ongoing transmission and allow for effective monitoring of AMR.

### New national initiatives

In addition to the efforts of front-line professionals, PHAC has put in place several initiatives to further improve the understanding and current levels and trends of AMR-GC infection in Canada and to provide better evidence to inform the development of treatment guidelines and public health interventions.

In 2013, the Enhanced Surveillance of Antimicrobial Resistant Gonorrhea was launched in several jurisdictions. This enhanced laboratory-epidemiological linked surveillance program collects information on demographics and clinical characteristics, risk behaviours, infection site(s), antimicrobial resistance and susceptibility, sequence typing and prescribed treatment information (53). Treatment data collected by this program in 2016 indicated that the majority of cases were prescribed either the preferred or alternative therapies as proposed by the CGSTI (4).

To support remote regions that cannot culture the GC isolates, the National Microbiology Laboratory (NML) has developed innovative technologies to detect and predict AMR directly from NAAT specimens (54–56). While it is important to note that these assays cannot replace culture-based determination of the minimum inhibitory concentration (MIC), they can still aid in surveillance by predicting antimicrobial susceptibilities of cephalosporin, ciprofloxacin and azithromycin and, together with molecular typing, can provide an understanding of the types of gonorrhea circulating within a community.

Finally, to support the monitoring of global patterns of AMR-GC, PHAC is engaged in international collaboration. National AMR surveillance data is submitted to the WHO Global STI Surveillance report and the Global Antimicrobial Resistance Surveillance System (GLASS). In an effort to standardize the characterization of gonococcal antimicrobial resistance genes, PHAC, in collaboration with researchers from Centers of Disease Control and Prevention (CDC), United Kingdom, Australia and Sweden, developed NG-STAR (*Neisseria gonorrhoeae* Sequence Typing for Antimicrobial Resistance) an on-line sequence based molecular antimicrobial resistance typing scheme for tracking the global dissemination of *N. gonorrhoeae* strains (57).

### Conclusion

Collaborative efforts between clinicians and public health professionals at local, provincial/territorial and federal levels are needed to effectively prevent, identify, treat and monitor GC and AMR-GC infections in Canada. There is no time like the present to hone our collective efforts to prevent emerging MDR-GC and XDR-GC strains from taking hold in Canada.
